# A robust and representative lower bound on object processing speed in humans

**DOI:** 10.1111/ejn.13100

**Published:** 2015-11-14

**Authors:** Magdalena M. Bieniek, Patrick J. Bennett, Allison B. Sekuler, Guillaume A. Rousselet

**Affiliations:** ^1^ Institute of Neuroscience and PsychologyCollege of Medical, Veterinary and Life SciencesUniversity of Glasgow58 Hillhead StreetGlasgowG12 8QBUK; ^2^ Department of PsychologyNeuroscience and BehaviourMcMaster UniversityHamiltonONCanada

**Keywords:** ageing, face processing, object processing, processing speed, visual evoked potentials

## Abstract

How early does the brain decode object categories? Addressing this question is critical to constrain the type of neuronal architecture supporting object categorization. In this context, much effort has been devoted to estimating face processing speed. With onsets estimated from 50 to 150 ms, the timing of the first face‐sensitive responses in humans remains controversial. This controversy is due partially to the susceptibility of dynamic brain measurements to filtering distortions and analysis issues. Here, using distributions of single‐trial event‐related potentials (ERPs), causal filtering, statistical analyses at all electrodes and time points, and effective correction for multiple comparisons, we present evidence that the earliest categorical differences start around 90 ms following stimulus presentation. These results were obtained from a representative group of 120 participants, aged 18–81, who categorized images of faces and noise textures. The results were reliable across testing days, as determined by test–retest assessment in 74 of the participants. Furthermore, a control experiment showed similar ERP onsets for contrasts involving images of houses or white noise. Face onsets did not change with age, suggesting that face sensitivity occurs within 100 ms across the adult lifespan. Finally, the simplicity of the face–texture contrast, and the dominant midline distribution of the effects, suggest the face responses were evoked by relatively simple image properties and are not face specific. Our results provide a new lower benchmark for the earliest neuronal responses to complex objects in the human visual system.

## Introduction

Visual processing speed is essential to constrain models of object processing, and their underlying architecture (Nowak & Bullier, 1997; Thorpe & Fabre‐Thorpe, 2001; Foxe & Simpson, 2002). In that context, much effort has been devoted to map the time course of object processing in animals and in humans, with a particular emphasis on brain responses to faces, and whether they support the existence of alternative face pathways (Cauchoix & Crouzet, 2013). In macaque monkeys, several studies have reported face selective responses at around 60–100 ms after stimulus onset (Sugase *et al*., 1999; Keysers *et al*., 2001; Edwards *et al*., 2003; Hung *et al*., 2005; Kiani *et al*., 2005; Afraz *et al*., 2006; Issa & DiCarlo, 2012), suggesting that 60 ms might be a lower bound for face detection in the macaque brain. In humans, the onset of face processing is less clear, with magnetoencephalography (MEG), scalp and intracranial electroencephalography (EEG) studies reporting face response onsets ranging from roughly 50–150 ms (e.g. Rousselet *et al*., 2008; Rossion & Caharel, 2011). For instance, several recent M/EEG studies have reported face sensitivity around or before 100 ms (Liu *et al*., 2009; Dering *et al*., 2011; Carlson *et al*., 2013; van de Nieuwenhuijzen *et al*., 2013; Cauchoix *et al*., 2014; Isik *et al*., 2014). Supporting these reports, it seems that some areas of the face network are active around 100 ms, as suggested by simultaneous EEG/functional magnetic resonance imaging (fMRI) and transcranial magnetic stimulation (TMS) studies (Pitcher *et al*., 2007, 2009; Sadeh *et al*., 2010). Thus, it is plausible that face‐sensitive responses occur before 100 ms in humans and monkeys. However, results from most human studies should be considered cautiously, because of several important limiting factors (Rousselet & Pernet, 2011; VanRullen, 2011; Rousselet, 2012). Most notably, the field is dominated by group analyses, with no quantification of onsets from individual participants. Too often, these group analyses are performed in time‐windows of interest, thus forfeiting onset quantification. Some studies use high‐pass filter settings that could lead to artificially early onsets (Luck, 2005; Acunzo *et al*., 2012; Rousselet, 2012; Widmann & Schroger, 2012; Widmann *et al*., 2015). Multiple comparisons over electrodes and time‐points are too often ignored or corrected using out‐dated methods (Pernet *et al*., 2015). Also, a small sample size of exclusively young adults is the norm, potentially providing an inaccurate estimation of population variability. Finally, a typical face experiment in humans lacks test–retest assessment.

The goal of the current study was to provide a lower bound on object processing in humans, using faces as a case study, while overcoming some of the limitations of previous research. To achieve this goal, we quantified onsets for arguably the simplest contrast between event‐related potentials (ERPs) to faces and noise textures lacking any object structure. We estimated these onsets in a large group of adult participants from a representative age distribution. Many of these participants were tested twice for test–retest reliability assessment of the results. We estimated onsets in each participant from data filtered to avoid potential onset distortions.

## Materials and methods

### Participants

In this study we pooled together data from 120 healthy participants (60 females) aged 18–81 years, recruited and tested in Canada (group 1: *n* = 30) and in the UK (group 2: *n* = 31, group 3: *n* = 59). Basic information about the participants is given in Table 1 and detailed descriptions are provided in Rousselet *et al*. (2009) for group 1, Rousselet *et al*. (2010) for group 2 and Bieniek *et al*. (2013) for group 3. A total of 74 participants took part in a second experimental session to assess the reliability of their results (24 participants from group 2 and 50 participants from group 3). The McMaster University research ethics board and the Glasgow University School of Psychology ethics committee approved the experiments. All participants provided written informed consent.

**Table 1:** Participants' information: for each age bracket the median age, years of education, visual acuity (measured using a Colenbrander high contrast card at 63 cm), and Pelli–Robson contrast sensitivity are given with minimum and maximum values in brackets

Age bracket (years)	Age (years)	No. of participants (females, males)	Years of education	Visual acuity	Contrast sensitivity
18–19	19 (18, 19)	6 (4, 2)	15.5 (15, 18.5)	1.25 (1, 1.6)	1.95 (1.95, 1.95)
20–29	22 (20, 29)	29 (14, 15)	18 (15, 25)	1.25 (0.8, 1.68)	1.95 (1.8, 2.25)
30–39	33 (30, 38)	15 (5, 10)	19 (14,25)	1.25 (0.8, 1.6)	1.95 (1.95, 2.1)
40–49	43.5 (40, 49)	16 (10, 6)	18 (12, 27)	1.25 (0.8, 1.6)	1.95 (1.95, 2.25)
50–59	55 (50, 59)	9 (3, 6)	19 (13, 19)	1.25 (0.63, 1.6)	1.95 (1.95, 1.95)
60–69	66 (60, 69)	31 (16, 15)	16 (5, 21.5)	0.96 (0.4, 1.39)	1.95 (1.95, 1.95)
70–81	73.5 (70, 81)	14 (8, 6)	13.5 (10, 21)	1 (0.4, 1.25)	1.95 (1.65, 1.95)

### Design and procedure

Participants from all three groups viewed images of faces (F) and textures (T). The same set of ten faces was used across the three experiments and is described in Gold *et al*. (1999). In short, all faces were front view greyscale images, cropped into an oval shape to remove external features (hair, ears). Textures were images with random Fourier phase spectra. All faces and textures had their Fourier amplitude spectra set to the average of the ten face amplitude spectra. All images were 256 × 256 pixels (visual angle: 8° × 8° for group 1 and 9° × 9° for groups 2 and 3). In the original studies, image Fourier phase coherence or screen luminance was manipulated (see illustrations in Rousselet *et al*., 2009; Bieniek *et al*., 2013). Thus, from each study, we chose trials from the conditions in which participants experienced stimuli with comparable Fourier phase coherence and screen luminance: group 1 – phase coherence = 70 and 0%, luminance = 33 cd/m^2^ (120 trials per condition); group 2 – phase coherence = 70–75% (pooled) and 0–5% (pooled), luminance = 33 cd/m^2^ (128 trials per condition); group 3 – phase coherence = 70 and 0%, luminance = 60.8 cd/m^2^ (150 trials per condition). For group 3, we chose the condition with luminance = 60.8 cd/m^2^ because the 31 cd/m^2^ condition had only 75 trials per condition and we found no difference in processing speed between 60.8 and 31 cd/m^2^ (Bieniek *et al*., 2013). We also found no ERP difference between 70 and 75% phase coherence, or between 0 and 5% (Rousselet *et al*., 2009, 2010). Groups 1 and 2 performed a one‐interval, two‐alternative forced choice task discriminating between two faces that included varying amounts of phase noise. On each trial, one face appeared briefly (53 ms), and participants had to indicate which of two possible faces was presented by pressing 1 or 2 on the numerical pad of the keyboard. Each participant performed the task with a single pair of male or female faces throughout the experiment. Group 3 had to discriminate between pictures of face and noise stimuli presented for 104 ms. They indicated their response by pressing 1 for face or 2 for noise on the numerical pad of the keyboard. In the three groups, participants were given unlimited time to respond, and were told to emphasize response accuracy, not speed. The task differences among studies should not affect onsets because task effects on face ERPs are weak to non‐existent within 200 ms (Rousselet *et al*., 2011). There were no onset differences among the three groups of participants in session 1 (one‐way anova:* F*_2,117_ = 0.26, *P* = 0.77), and between the two groups of participants in session 2 (*F*_1,72_ = 0.21, *P* = 0.65).

### EEG data pre‐processing

EEG data were obtained in Canada using a 256‐channel Geodesic Sensor Net (Electrical Geodesics Inc., Eugene, OR, USA), and in the UK using a Biosemi Active Electrode Amplifier System with 128 electrodes. Data were pre‐processed using Matlab 2012a and EEGLAB 11.0.2.1b (Delorme *et al*., 2011). Data were first re‐referenced off‐line to an average reference. Subsequently, the same data were filtered in two different ways. First, to measure onsets, we used a 2‐Hz causal fourth‐order Butterworth high‐pass filter to avoid onset distortion associated with non‐causal filtering. Second, we used a 1‐Hz non‐causal fourth‐order Butterworth high‐pass filter to perform independent component analysis (ICA). Due to high levels of power line noise, the Canadian dataset (group 1) was also low‐pass filtered using a 30‐Hz non‐causal fourth‐order Butterworth filter. Subsequently, all datasets were re‐sampled at 500 Hz and epoched between −300 and 1000 ms around stimulus onset. In the causal filtered dataset, baseline correction was performed using the average activity between time 0 and −300 ms, whereas in the non‐causal filtered dataset, individual channel mean was removed from each channel, which increases ICA reliability (Groppe *et al*., 2009). Noisy electrodes were identified by visual inspection of the non‐causal filtered data and rejected from the causal and non‐causal datasets. ICA was performed on the non‐causal filtered data using the infomax algorithm as implemented in EEGLAB. Components representing blinks were then identified and removed from both causal and non‐causal filtered datasets (number of ICs removed: median = 2, min. = 0, max. = 10). Subsequently, data were re‐epoched between −300 and 600 ms and baseline correction was performed again. Finally, data epochs were removed based on an absolute threshold value larger than 100 μV and the presence of a linear trend with an absolute slope larger than 75 μV per epoch and *R* ^2^ larger than 0.3. Across participants, the median number of trials available for analyses was, for faces (session 1/session 2): 127/146; min. = 27/92; max. = 150/150; for textures: 127.5/146; min. = 25/91; max. = 150/150. There was no significant relationship between the number of face/texture ERP trials and ERP face sensitivity onsets.

### EEG data analysis

**Figure 1:**
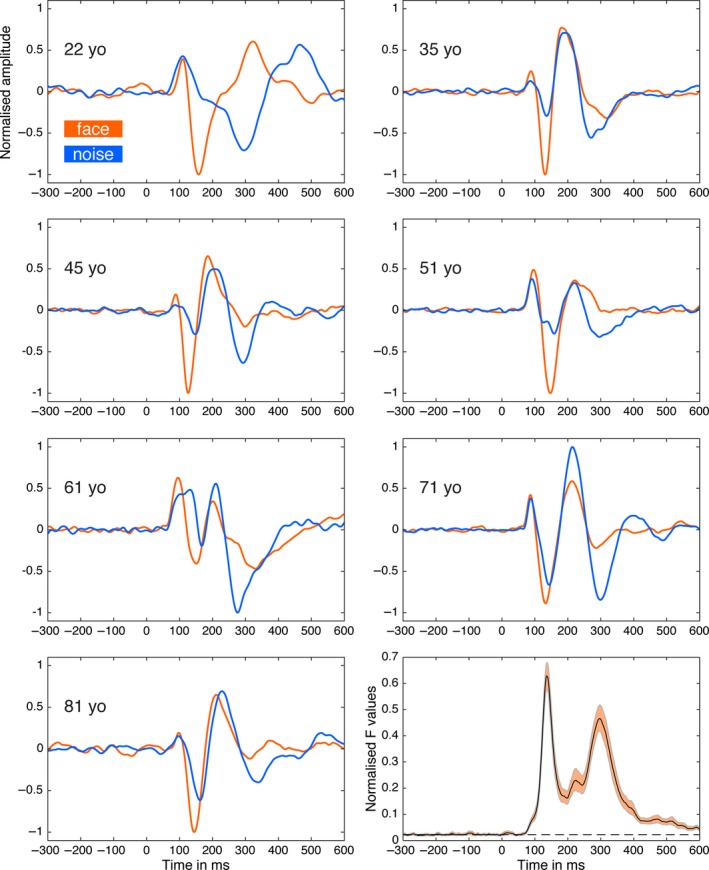
ERP results. For different age groups, we illustrate examples of ERPs to face (orange) and noise (blue) stimuli at the electrode with the maximum absolute *t* value. The age of each participant is reported in each plot. The lower right plot shows the time‐course of the mean across all participants of the maximum *F* values (squared *t* values) across all electrodes in session 1. *F* values were normalized [0, 1] before averaging. The shaded area shows the 95% percentile bootstrap confidence interval. The *F* values depart from baseline shortly before 100 ms.

Statistical analyses were conducted using Matlab 2012a and the LIMO EEG toolbox (Pernet *et al*., 2011). Because we used causal filters, a legitimate concern is whether effects from one trial could be smeared forward in time to the next trial. This was not the case, as illustrated in Fig. 1, which shows flat baselines in a sample of participants with large ERP effects that would be most likely to introduce forward distortions.

### Single participant data analyses

To determine the onset of face ERP sensitivity, in every participant we computed *t*‐tests between face and texture ERPs. All single‐participant tests were performed independently at every electrode and every time point. We controlled for multiple comparisons by using a bootstrap spatial–temporal clustering approach (Maris & Oostenveld, 2007; Pernet *et al*., 2011, 2015). The onset of ERP face sensitivity was defined independently in each participant as the first significant *t*‐test across all electrodes, after removing any significant cluster that started before stimulus onset (which happened in three participants).

We compared onsets obtained using *t*‐tests with standard means (*mean* data) against those obtained with 20% trimmed means (*tmean* data). *t*‐Tests on 20% trimmed means can help increase power and might reveal earlier onsets in noisier EEG sessions (Rousselet *et al*., 2008; Wilcox, 2012; Desjardins & Segalowitz, 2013). We compared mean with trimmed mean onsets using data without low‐pass filtering (*mean* vs. *tmean*), and after application of a low‐pass filter (*mean lp* vs. *tmean lp*) to check if low‐pass filtering, as commonly applied in ERP research, produces signal distortions leading to artificially earlier onsets (VanRullen, 2011). This comparison was performed in 90 participants only, because 30 participants from the Canadian dataset had to be low‐passed filtered during the pre‐processing stage to reduce line‐noise. Low‐pass filtered ERP data deposited in the Dryad repository: http://dx.doi.org/10.5061/dryad.46786.

### Effect size

For each participant, we computed effect sizes at the time of ERP face sensitivity onsets for *mean*,* tmean*,* mean lp* and *tmean lp* data. We used a non‐parametric measure of effect size, Cliff's *delta* (Cliff, 1996; Wilcox, 2006), which is related to the Wilcoxon–Mann–Whitney *U* statistic and estimates the probability that a randomly selected observation from one group is larger than a randomly selected observation from another group, minus the reverse probability. Cliff's *delta* ranges from 1 when all values from one group are higher than the values from the other group to −1 when the reverse is true. Completely overlapping distributions have a Cliff's *delta* of 0.

### Group analyses

Group analyses were performed on onsets obtained from the single participant data analyses. Data and code deposited in the figshare repository: http://dx.doi.org/10.6084/m9.figshare.1588513. We quantified distributions of onsets using the Harrell–Davis estimate of the deciles (Wilcox, 2012). Throughout the article, square brackets report 95% bootstrap confidence intervals with 1000 samples. We used a *shift function* to quantify differences between deciles of onset distributions (Doksum, 1974, 1977; Wilcox, 2006). We computed group‐level regressions using Matlab's *robustfit* function, with default parameters. We report slopes and intercepts along with 95% percentile bootstrap confidence intervals.

### Control experiment

So far, we have described parametric analyses of univariate differences in means and trimmed means between distributions of single‐trial face and noise ERPs. But single‐trial ERP distributions could in principle differ not only in central tendency, but also in dispersion, skewness and kurtosis. Differences between ERP conditions could also be distributed across electrodes. Our tests of central tendency would not be sensitive to such differences and therefore might miss earlier onsets. In addition, we wanted to determine if the face onsets measured in the main experiment were comparable to onsets involving other image categories. To address these issues, we re‐analysed ERP data from an experiment in which eight observers categorized pictures of faces, houses and noise textures, presented for 53 ms (Bieniek *et al*., 2012). Seven observers were tested twice. There were up to 1000 trials per observer in total: 300 face trials, 300 house trials, 300 trials of phase noise textures with the same amplitude spectra as faces and houses, and 100 white noise trials. EEG preprocessing was as described above, except that the causal filtered data used to measure onsets were not low‐pass filtered, but were transformed into single‐trial spherical spline current source density waveforms using the current source density (CSD) toolbox (Tenke & Kayser, 2012). The CSD transformation is a spatial high‐pass filtering of the data, which sharpens ERP topographies and reduces the influence of volume‐conducted activity. We hoped this transformation would help to identify earlier effects by de‐blurring scalp ERPs. CSD waveforms were computed using 50 iterations and the parameters *m* = 4, lambda = 10^−5^.

We applied several statistical tests to measure onsets defined by differences between pairs of conditions: linear contrasts (*t*‐tests) on means and 20% trimmed means, two‐sample Kolmogorov–Smirnoff tests, mutual information and logistic regression. Mutual information is a non‐parametric estimate of the dependence, whether linear or non‐linear, between pairs of variables (Ince *et al*., 2010). We calculated mutual information using the direct method, quadratic extrapolation bias correction and four equiprobable bins (Magri *et al*., 2009). We searched for effects distributed across electrodes using logistic regression, independently at each time point, with a one time‐point training window, and leave‐one‐out cross validation (Philiastides & Sajda, 2006). We report results without classifier regularization, which performed very well: in every participant and session, at least one contrast gave classification performance > 90%, and all baselines were centred on 50%. Reducing the number of trials also had little effect on classification accuracy, suggesting that performance had reached an asymptote. We also used l2 regularization (Conroy & Sajda, 2012), with λ = 0.1 optimized for maximum classification performance across time points. With regularization, onsets were only 2.2 ms [0.8, 4.5] earlier than without regularization.

Onsets were measured using bootstrap clustering techniques with 1000 bootstrap samples except for logistic regression, for which we used 200 bootstraps because it was extremely time‐consuming. In the Results, we report, for every statistical test, the minimum onset across the two sessions for the seven participants tested twice, and the single onset from participant 8.

## Results

**Figure 2:**
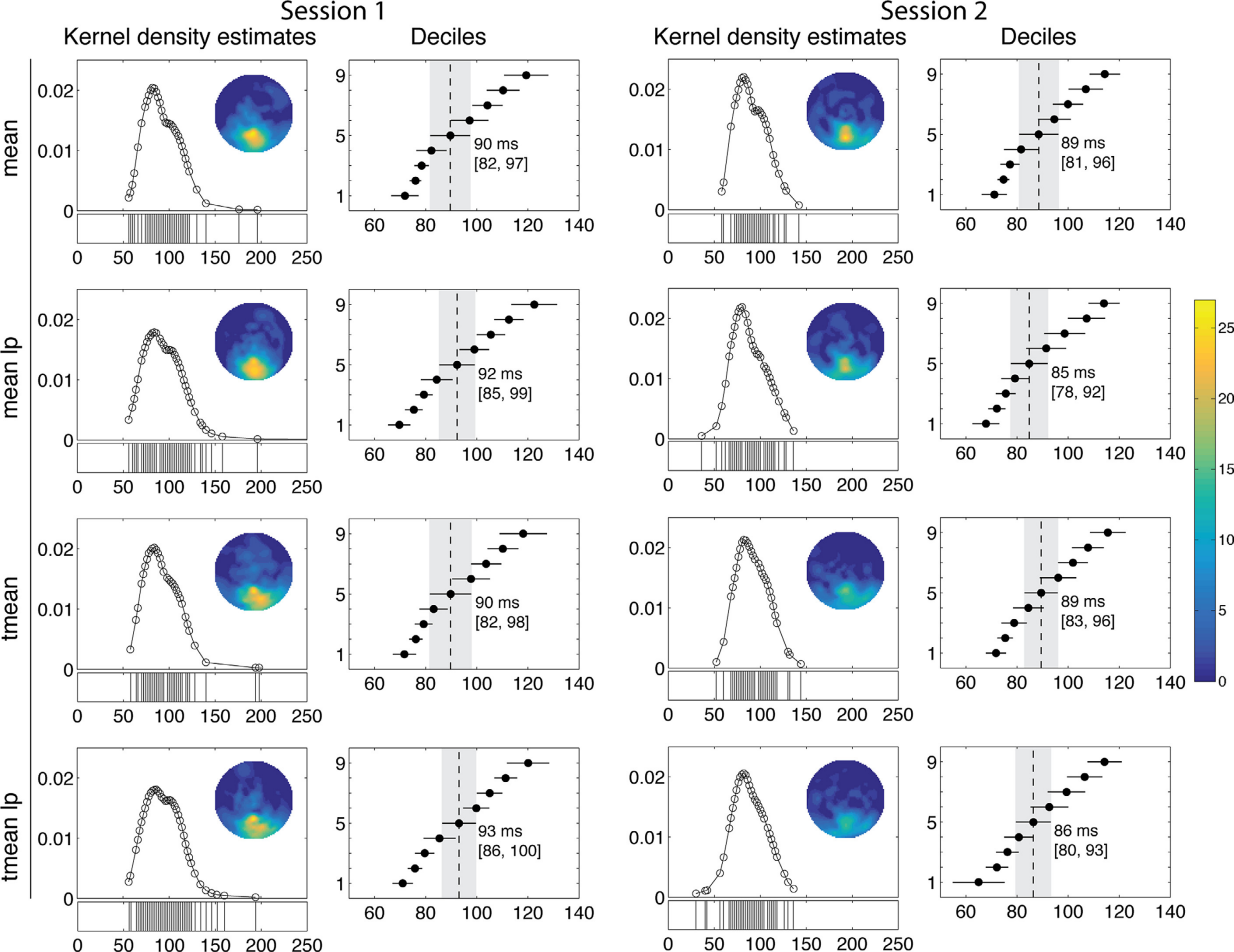
Onset distributions. Results are shown for each of the four conditions (mean, tmean, mean lp and tmean lp) in session 1 and session 2. Columns 1 and 3 show the kernel density estimates of onset distributions. The circles on each plot indicate the estimated onset frequencies. Horizontal plots beneath the kernel density estimates (KDEs) depict individual participants' onsets. Columns 2 and 4 show the onset deciles with 95% confidence intervals (CIs). The vertical dashed line in each plot marks the median and the shading highlights the boundaries of its 95% CI, which is also given in square brackets. Topographic maps show how many participants had onsets at each scalp location, with a scale described in the far right colour bar.

**Figure 3:**
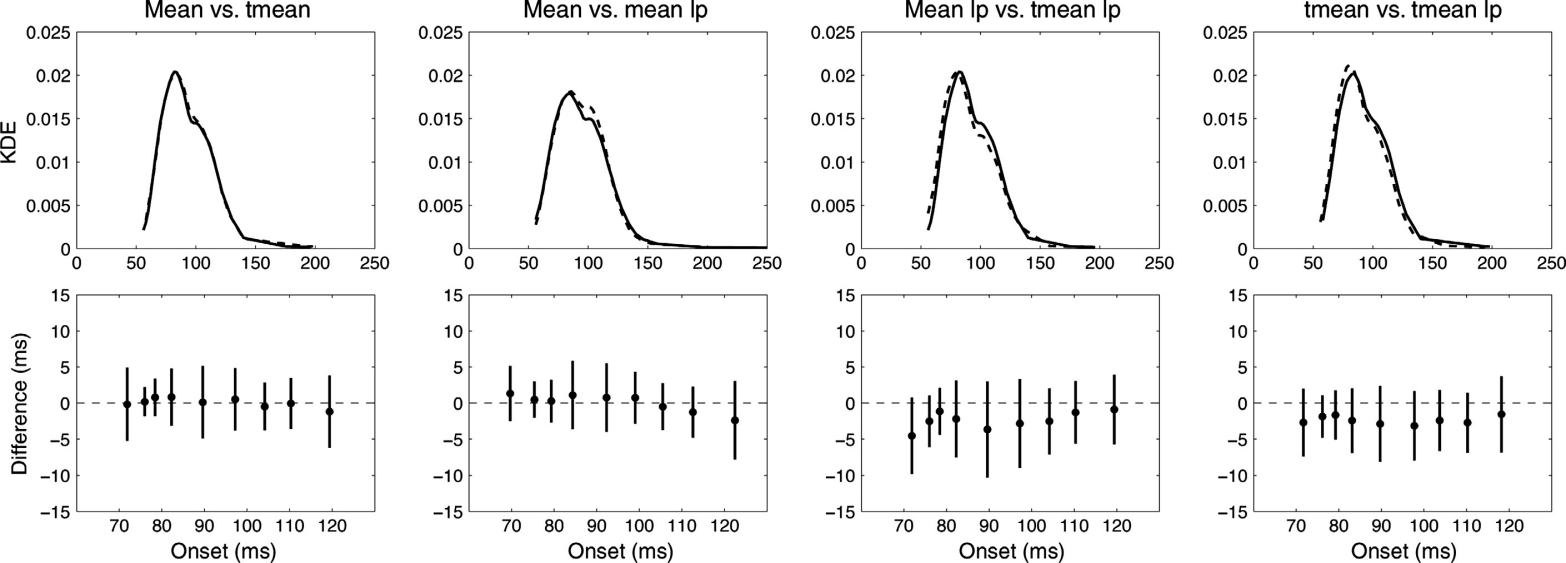
Comparisons of onset distributions in session 1. The first row shows the KDEs of pairs of conditions: the continuous line represents condition 1 from the title (e.g. mean in column 1), and the dashed line represents condition 2 (e.g. tmean). The second row shows shift functions. Shift functions allow systematic comparisons of two distributions. The *x*‐axis shows the onset deciles for the first group of the comparison (e.g. mean in the mean vs. tmean comparison). The *y*‐axis shows the differences between onset deciles from the two groups (filled circles). The vertical lines indicate the 95% CIs of the onset differences. The results indicate no significant difference in any part of the onset distributions. Similar results were observed in Session 2.

Consistent with recent EEG and MEG findings, our results suggest the existence of neuronal face sensitivity within 100 ms (Figs 1 and 2). Using *t*‐tests with means on low‐pass filtered data revealed a median onset of 92 ms [85, 99] in session 1 (Fig. 2, mean lp data). The 1st decile of the distribution was 70 ms [65, 74] and the 9th decile was 122 ms [113, 131]. These estimates did not change significantly for data that were not low‐pass filtered, or when 20% trimmed means were used instead of means (Figs 2 and 3). Because there were no significant differences between onset distributions in any of the comparisons (Fig. 3), all further analyses were only performed on the *mean lp* data.

**Figure 4:**
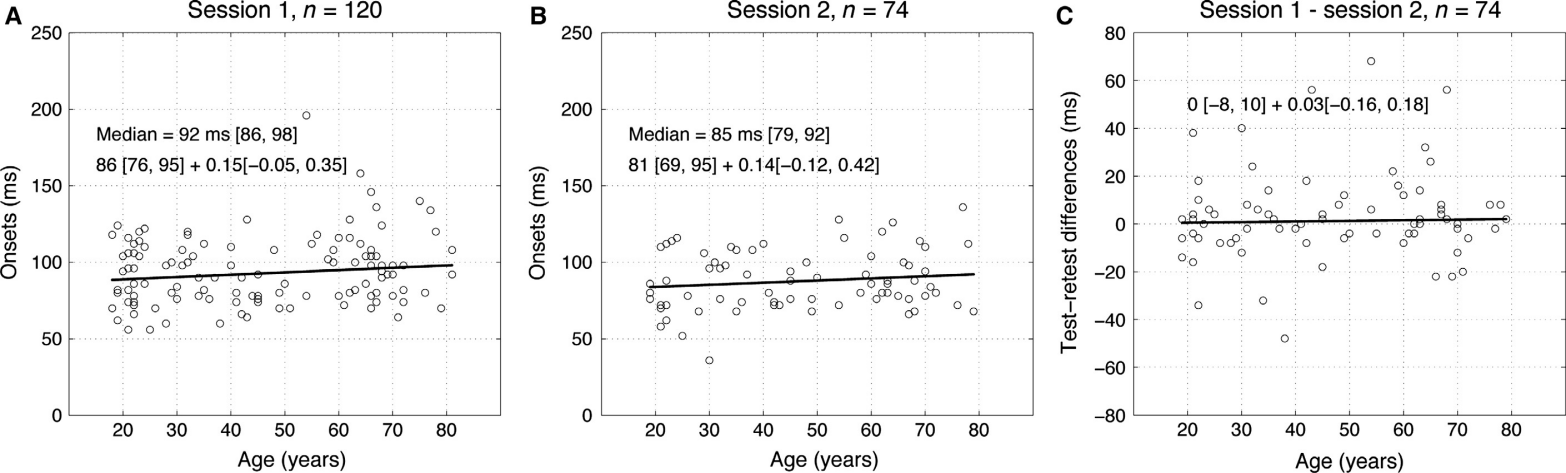
Regressions of onsets against age for mean lp data. Each circle represents the onset from one participant. The regression line appears in black. (A,B) Results from sessions 1 and 2. The group median onset and its 95% CI is indicated at the top of each scatterplot. Beneath, the equation describes the regression intercept and slope and their respective 95% CIs. (C) Results for the between‐session onset differences. In each case, the dependent variable was not significantly associated with age.

Our data were from participants 18–81 years old. Thus, we looked at the relationship between ERP face sensitivity onsets and age, and found no evidence for a significant relationship (Fig. 4A and B). This finding is consistent with previous observations of ageing affecting the time‐course of face ERPs starting around 120 ms after stimulus onset, thus sparing the earliest face responses (Rousselet *et al*., 2010; Bieniek *et al*., 2013). We also performed a multiple regression analysis to test if onset variability could be explained by participants' age, visual acuity, contrast sensitivity, years of education or sex. None of these variables was significantly associated with onset times.

**Figure 5:**
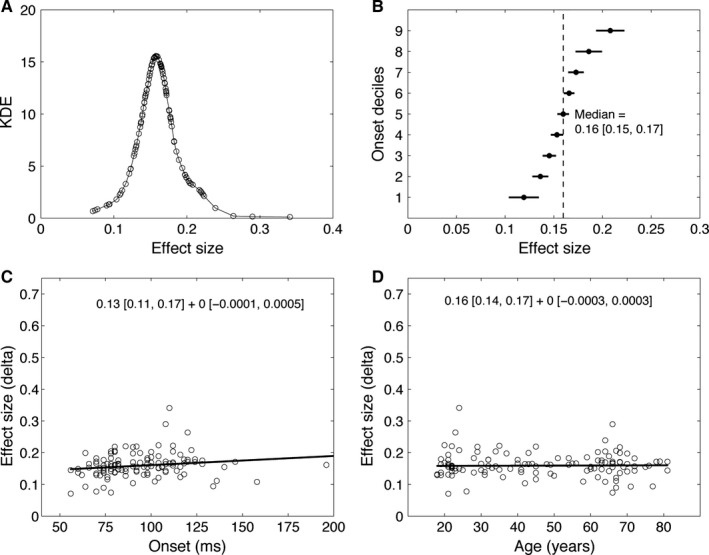
Effect sizes at onset times. (A) KDEs of effect size distributions. (B) Deciles of effect size distributions with 95% CIs. The median is marked by a vertical dashed line. (C) Linear regression of effect size on onset. (D) Linear regression of effect size on age. In C and D, each circle represents a participant. The regression equation with intercept and slope and their corresponding 95% CIs are given at the top of each scatterplot. All results are from session 1 (*n* = 120). Similar results were observed in session 2.

Next we looked at how big the effects were at ERP onset times (Fig. 5). In the majority of participants, Cliff's *delta* estimates of effect sizes ranged from 0.1 to 0.3, which corresponds to small to medium effect sizes in Cohen's *d* framework. There was no significant relationship between onset latencies and effect sizes (Fig. 5C), which means that later onsets were not systematically associated with smaller (or larger) effect sizes compared with earlier onsets. Effect sizes also did not depend on participants' age (Fig. 5D), suggesting that ageing does not affect the size of ERP face differences at onset time, contrary to what happens beyond 120 ms post‐stimulus onset (Rousselet *et al*., 2010; Bieniek *et al*., 2013).

**Figure 6:**
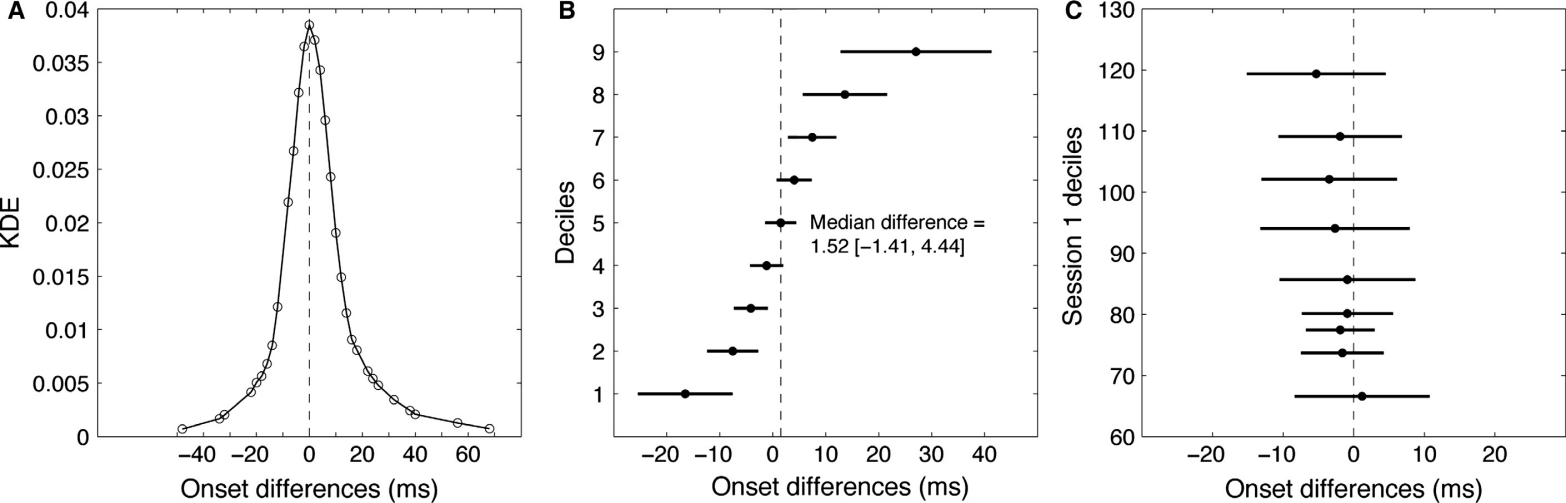
Test–retest differences. (A) KDE of the distribution of pairwise onset differences between sessions. (B) Deciles of the test–retest differences. (C) Shift function. Filled circles represent differences in onset times between sessions (*x*‐axis), at each decile of session 1 onsets (*y*‐axis); horizontal lines mark the boundaries of the 95% CIs around these differences. The shift function suggests the lack of significant between‐session differences, whether onsets were early or late in session 1.

Event‐related potentials onsets were reliable (Fig. 6). The distribution of test–retest differences is symmetric and centred on zero, indicating no systematic bias across sessions (for instance onsets could have been systematically earlier in session 2). Also, across the 74 participants tested twice, no significant differences were found between any of the onset deciles (Fig. 6C). This last result is important because it demonstrates that test–retest reliability does not depend on onset times. One could have imagined for instance that the earliest onsets might have been obtained by chance, so that a second test would be systematically biased towards longer onsets: our analysis suggests that this was not the case. Finally, test–retest differences were not significantly associated with participants' age (Fig. 4C), suggesting the absence of age‐related differences in processing speed reliability.

So far, our analyses were applied to the full sample of 120 participants in session 1, and 74 participants in session 2. This sample size is larger than samples used in most ERP studies, and therefore it is worth considering what could be expected given fewer participants. To answer this question, we estimated the variability in median onsets as a function of sample size using Monte‐Carlo simulations. To this end, we drew samples of 5–70 participants, in steps of five. For each sample size, participants were sampled with replacement 10 000 times, and every time the median onset and the median of the between‐session onset differences were computed.

**Figure 7:**
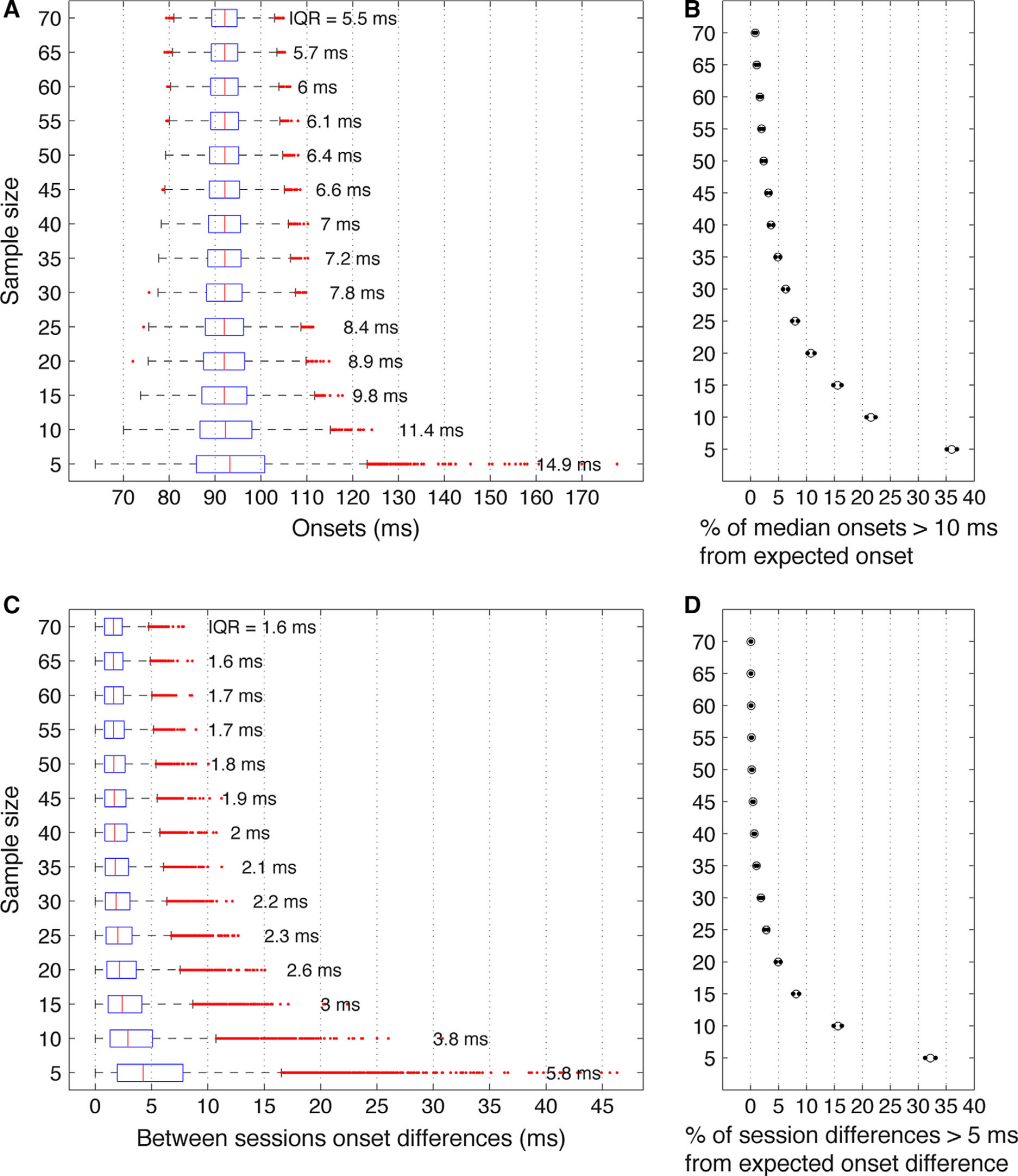
Monte‐Carlo estimates of median onsets and median onset differences between sessions as a function of sample size. In all panels, the *y*‐axis depicts sample size, from five to 70 participants. (A) Boxplots of median onset estimates. The red vertical line within each boxplot indicates the median, the extremities of the blue box indicate lower and upper quartiles, and whiskers extend to the most extreme data points, not including outliers, which are marked individually by red dots. (B) Proportions of median onsets at least 10 ms larger than the expected median onset from the full sample. (C) Boxplots of medians of absolute between‐session onset differences. (D) Proportions of median absolute onset differences at least 5 ms larger than the expected median difference.

Medians of Monte‐Carlo estimates of onsets changed very little as a function of sample size (Fig. 7A). However, smaller sample sizes were associated with much larger variability, particularly on the right side of the median distribution. This right skewness implies that, in the long run, testing too few participants would lead to over‐estimating onsets, as illustrated in Fig. 7B. Testing at least 20 participants appears to reduce this problem considerably.

Similarly to the median estimates, the medians of the between‐session differences were fairly stable across sample sizes. The distributions of between‐session differences were also right skewed, and this effect was considerably increased at the smallest sample sizes. The consequence of this asymmetry is a long‐run tendency to over‐estimate between‐session differences. It seems that testing at least 20 participants would considerably improve the test–retest reliability of the median estimates.

### Control experiment results

**Figure 8:**
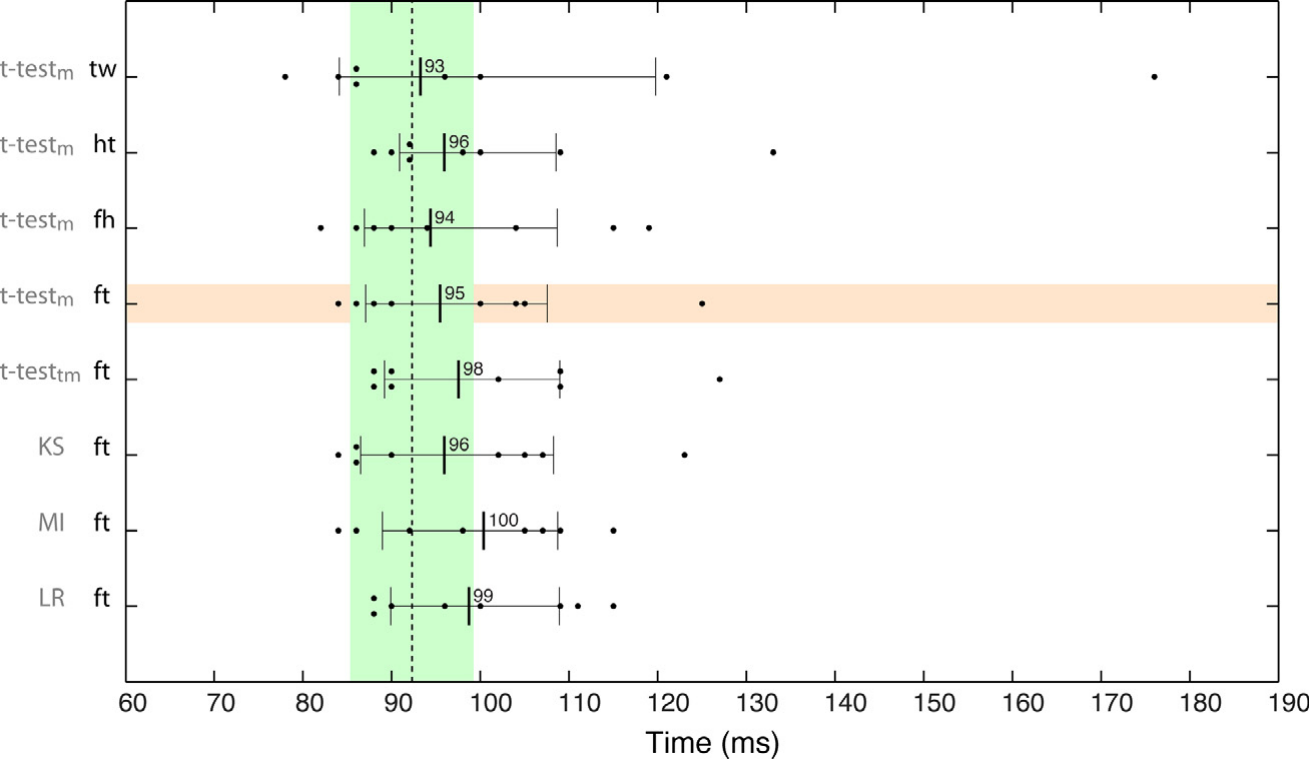
Onsets from the control experiment. The scatterplots show the onsets of eight participants, in five families of statistical tests. For the seven participants who were tested twice, each black dot represents the minimum onset across sessions for each test. For each test, the vertical thick line and the number next to it indicate the median onset across participants. The thin vertical lines mark the boundaries of the median's 95% CI. The long vertical dashed line marks the median onset from session 1 of the main experiment, in condition mean lp for the face–texture contrast. The green area marks that median's 95% CI. The horizontal orange area marks the matching results from the control experiment. Abbreviations: tw, texture – white noise contrast; ht, house – texture contrast; fh, face – house contrast; ft, face – texture contrast; *t*‐testm, *t*‐test on means; *t*‐testtm, *t*‐test on 20% trimmed means; KS, Kolmogorov–Smirnov test; MI, mutual information; LR, logistic regression.

The goal of the control experiment was to test in an independent dataset (*n* = 8) if any earlier onsets could have been missed because of differences not captured by parametric comparisons of central tendency. We also tested whether similar onsets would be observed for a control object category (houses) and between our structured textures and a control texture (white noise). For each of eight participants – seven of whom were tested twice for a total of 15 EEG sessions – we looked for differences in mean, variance, skewness and kurtosis between four pairs of image categories: textures vs. white noise; faces vs. textures; houses vs. textures; faces vs. houses. Within 200 ms after stimulus onset, only pairwise comparisons between means were significant. Corroborating this result, in every participant and for each pairwise comparison, kernel density estimates and shift functions revealed that at onset times, the shapes of the single‐trial ERP distributions were similar across stimulus categories. ERP differences between pairs of image categories were due to shifts of entire distributions, which should be well captured by quantifying differences between means. We confirmed that categorical ERP differences were essentially due to differences in means during the first 200 ms after stimulus onset by estimating ERP category onsets using a range of robust and non‐parametric techniques (Fig. 8). Onsets derived from *t*‐tests on means were similar to those reported in the main experiment. Results from the other methods were very similar, and all the pairwise comparisons between onsets from the main experiment and from the control experiment failed to find significant differences, even without controlling for multiple comparisons. Thus, the results from the control experiment suggest that *t*‐tests on the mean, combined with modern control for multiple comparisons, are adequate and sufficient to capture early differences in single‐trial ERP distributions.

## Discussion

Estimating processing speed is a critical step to constrain the type of neuronal architecture underlying cognitive processes. This approach has been particularly fruitful to study visual processing, revealing the hierarchical structure of the visual system (Mormann *et al*., 2008; DiCarlo *et al*., 2012). Despite recent advances, in humans there is still considerable debate about the timing of object processing, and especially of face processing. To address this controversy, we measured ERP onsets of face sensitivity in a sample of 120 healthy participants, 18–81 years old. Across participants, median onset was 92 ms [85, 99], with the 1st decile at 70 ms and the 9th decile at 122 ms. To our knowledge, the current study is the first to report a detailed distribution of onsets from such a large sample size. This is in contrast to most previous studies, which have limited their analyses to time‐windows of interest, without quantifying onsets *per se* (e.g. Liu *et al*., 2002; Thierry *et al*., 2007). Several recent studies have estimated onsets using group statistics, thus providing a single group estimate, without a confidence interval (Liu *et al*., 2009; Carlson *et al*., 2013; van de Nieuwenhuijzen *et al*., 2013; Cauchoix *et al*., 2014; Isik *et al*., 2014; Clarke *et al*., 2015). Our onset estimations are also unlikely to have been biased by filtering distortions, or inefficient control for multiple comparisons. Previous studies have mostly used potentially ineffective control for multiple comparisons in which an effect is considered significant if it is sustained for an a‐priori determined number of time points (Piai *et al*., 2015), or they have used conservative maximum statistics, or other techniques that fail to consider the spatial–temporal distributions of the effects (Pernet *et al*., 2015). Also, some highly cited studies have employed 1‐Hz high‐pass filters (e.g. Liu *et al*., 2002, 2009), which can smear effects back in time (Rousselet, 2012). For instance, Liu *et al*. (2009), among other results from an ambitious intracranial study, reported a rare distribution of onsets for object classification, including faces, starting around 30 ms after stimulus presentation (their Fig. 3A). Our results call into question such reports of very early face sensitivity under 70 ms. Our results are nevertheless compatible with the timing from many previous reports listed above. And these studies are certainly not the first to report evidence for face processing within 100 ms (see, for example, reviews by Rousselet *et al*., 2008; Rossion & Caharel, 2011).

So how long does it take to detect a face? Our results can only provide a lower bound to that question. Indeed, our choice of a simple face–texture contrast implies that any local edge could be responsible for our ERP onsets, so there is no ground to argue the face‐specificity of the effects reported here. This choice was intentional as we wanted to quantify the earliest possible face responses. And several elements of our results suggest that we did not miss earlier effects. First, we found no evidence that our techniques lacked power, because we obtained very similar results using *t*‐tests on means or trimmed means. Second, results from a control experiment suggested that categorical ERP differences at onset time essentially reflect shifts in the means of single‐trial distributions, so that statistical tests sensitive to other distributional differences other than central tendency did not reveal earlier onsets. Furthermore, there was no relationship between effect sizes and ERP onsets, suggesting that the large individual differences in onset times were not associated with systematic differences in signal‐to‐noise ratio across participants. Onset estimates were reliable across two testing days, with only about 5–10 ms difference between sessions, which is consistent with studies showing stability of scalp and intracranial ERPs across hours and days (Bansal *et al*., 2012; Hammerer *et al*., 2013). Finally, the timing of our effects and their scalp distribution suggest the involvement of relatively low‐level areas sensitive to coarse image properties (Tanskanen *et al*., 2005; Scholte *et al*., 2009; Groen *et al*., 2012, 2013; Cauchoix *et al*., 2014; Rousselet *et al*., 2014), a conclusion supported by the similar latencies observed for several contrasts among object categories in our control experiment. The involvement of relatively low‐level areas is also supported by the small timing difference between our earliest onsets (around 70 ms) and onsets to simple stimuli around 50–60 ms from striate and extra‐striate areas (Foxe & Simpson, 2002). However, we cannot completely rule out the contribution of higher‐level areas from the face network, which is activated within 100 ms (Barbeau *et al*., 2008; Liu *et al*., 2009; Parvizi *et al*., 2012; Jonas *et al*., 2014; Pitcher, 2014). Given the low spatial resolution of EEG, we do not see the benefit of performing source analysis on our current dataset: instead, large‐scale MEG or combined TMS/EEG‐fMRI studies would be more informative.

How the onsets reported here relate to behaviour remains unknown. In our experiment, participants were asked to be as accurate as possible, with no emphasis on speed, so we did not attempt a reaction time analysis. Certainly, behavioural reaction times can be used to estimate relative processing durations, and would provide useful upper bounds on processing speed (VanRullen, 2011). But there might be no relationship between our onset estimates and behavioural responses, because behavioural variability could originate from neuronal variability outside the ventral pathway (DiCarlo & Maunsell, 2005; Gerson *et al*., 2005; Philiastides & Sajda, 2006).

Overall, the current study establishes a new lower benchmark for the earliest ERP responses to complex objects, including faces, in the human visual system. This benchmark is of course not absolute. We used heavily simplified greylevel cropped‐out faces, in full‐front position. The timing of early face differences should be systematically investigated using more realistic stimuli, with different sizes, positions and orientations. Also, we have previously demonstrated large effects of screen luminance on ERP latencies (Bieniek *et al*., 2013). It remains to be determined how fast the visual system can process real three‐dimensional stimuli under realistic daylight conditions, outside our typical testing booths, in which dark‐adapted participants look at bright screens. It is also likely that yet better EEG preprocessing and other brain imaging techniques could improve our current estimates. At least here we provide a framework to study ERP onsets.

Finally, our results suggest large individual differences in ERP onsets, which remain unexplained. We found that ERP onsets did not change with age, replicating previous observations and suggesting that ageing affects face processing beyond the earliest stage of image structure detection (Bieniek *et al*., 2013). ERP onsets were also not associated with visual acuity and contrast sensitivity. A challenge for the community would therefore be to quantify results from large samples of individual participants with the goal of understanding differences in processing speed (Rousselet *et al*., 2010). How many participants should be tested remains an open question and depends on the goal of the experiment. From our results, it seems that in the long run, testing at least 20 participants would help reduce the risk of over‐estimating onsets and would increase their reliability. However, this is certainly not sufficient to understand individual differences (Yarkoni, 2009).
